# Disruption of RNA Splicing Increases Vulnerability of Cells to DNA-PK Inhibitors

**DOI:** 10.3390/ijms252111810

**Published:** 2024-11-03

**Authors:** Anastasia P. Kovina, Artem V. Luzhin, Victor V. Tatarskiy, Dmitry A. Deriglazov, Natalia V. Petrova, Nadezhda V. Petrova, Liya G. Kondratyeva, Omar L. Kantidze, Sergey V. Razin, Artem K. Velichko

**Affiliations:** 1 Department of Cellular Genomics, Institute of Gene Biology RAS, 119334 Moscow, Russia; anastasiyaa.kovina@gmail.com (A.P.K.); artyom.luzhin@gmail.com (A.V.L.); drglz.dm@gmail.com (D.A.D.); petrovanv@mail.ru (N.V.P.); petrova.nadezhda.v@gmail.com (N.V.P.); kantidze@gmail.com (O.L.K.); sergey.v.razin@inbox.ru (S.V.R.); 2 Center for Precision Genome Editing and Genetic Technologies for Biomedicine, Institute of Gene Biology RAS, 119334 Moscow, Russia; tatarskii@gmail.com; 3 Institute of Gene Biology RAS, 119334 Moscow, Russia; 4 Shemyakin-Ovchinnikov Institute of Bioorganic Chemistry, 117997 Moscow, Russia; liakondratyeva@yandex.ru; 5 Biological Faculty, Lomonosov Moscow State University, 119992 Moscow, Russia; 6 Institute for Translational Medicine and Biotechnology, Sechenov First Moscow State Medical University, 119991 Moscow, Russia

**Keywords:** DNA-PK, CRISPR/Cas9 screening, RNA splicing, NU7441, NU7026, pladienolide B

## Abstract

DNA-dependent protein kinase (DNA-PK) is a key effector of non-homologous end joining (NHEJ)-mediated double-strand break (DSB) repair. Since its identification, a substantial body of evidence has demonstrated that DNA-PK is frequently overexpressed in cancer, plays a critical role in tumor development and progression, and is associated with poor prognosis in cancer patients. Recent studies have also uncovered novel functions of DNA-PK, shifting the paradigm of the role of DNA-PK in oncogenesis and renewing interest in targeting DNA-PK for cancer therapy. To gain genetic insight into the cellular pathways requiring DNA-PK activity, we used a CRISPR/Cas9 screen to identify genes in which defects cause hypersensitivity to DNA-PK inhibitors. We identified over one hundred genes involved in DNA replication, cell cycle regulation, and RNA processing that promoted cell survival when DNA-PK kinase activity was suppressed. This gene set will be useful for characterizing novel biological processes that require DNA-PK activity and identifying predictive biomarkers of response to DNA-PK inhibition in the clinic. We also validated several genes from this set and reported previously undescribed genes that modulate the response to DNA-PK inhibitors. In particular, we found that compromising the mRNA splicing pathway led to marked hypersensitivity to DNA-PK inhibition, providing a possible rationale for the combined use of splicing inhibitors and DNA-PK inhibitors for cancer therapy.

## 1. Introduction

A DNA damage response (DDR) network encompasses multiple cellular pathways that sense, signal, and repair DNA damage, all of which are critical for cell survival and genome maintenance. Double-strand breaks (DSBs) are the most detrimental and toxic forms of DNA damage, that, if not repaired, lead to cell cycle arrest and cell death [1]. Two primary pathways are employed to repair DSBs: the high-fidelity homologous recombination (HR) pathway [2,3] and the less accurate non-homologous end joining (NHEJ) pathway [3,4]. Although both pathways ensure genome integrity in normal cells, cancer cells exploit DDR activation processes to acquire more aggressive phenotypes and develop resistance to chemotherapy [5]. Among the numerous DDR proteins that are dysregulated in cancer, the catalytic subunit of DNA-dependent protein kinase (DNA-PKcs, referred to here as DNA-PK) plays a pro-tumorigenic role in many types of cancer including prostate, breast, colorectal, and cervical cancers, as well as chronic leukemias [6,7,8]. In particular, DNA-PK activation increases with disease progression, whereas its elevated expression and activity correlate with resistance to both chemotherapy and radiotherapy, as well as with overall poor prognosis, making DNA-PK a promising oncotherapeutic target [9,10,11,12,13].

In addition to its well-established canonical role, DNA-PK also has a range of pleiotropic non-DDR cellular functions relevant to cancer. For example, it participates in the response to replicative stress [14], regulates autophagy [15], controls the cell cycle [16], is involved in ribosomal RNA biogenesis [17,18], and maintains telomere integrity [19]. Moreover, DNA-PK participates in the regulation of pro-metastatic gene transcription in certain cancer types, as well as the secretion of factors that modulate tumor cell invasion and metastasis [20]. Collectively, these data suggest that DNA-PK plays an important role in pathways beyond DDR that are essential for the survival and proliferation of cancer cells.

In this study, we sought to identify novel regulatory pathways associated with DNA-PK. We demonstrated that, in the absence of DSBs, the pharmacological inhibition of DNA-PK using laboratory-grade DNA-PK inhibitors (DNA-PKi–NU7441 or NU7026) blocked DNA replication in HT1080 and HeLa cells and suppressed these cells proliferation. Transcriptomic analysis revealed the modulation of pathways known to be regulated by DNA-PK, including cell cycle- and DNA replication-associated pathways. Additionally, new cancer-related processes modulated by DNA-PK were identified, including mRNA splicing. Finally, using genome-wide CRISPR/Cas9 screening, we identified over one hundred genes that promote cellular resistance to DNA-PKi. Specifically, we found that loss-of-function mutations in genes encoding regulators of mRNA processing and splicing confer DNA-PKi sensitivity. Consistent with this observation, the pharmacological inhibition of splicing with the spliceosome factor 3b (SF3b) inhibitor pladienolide B, in combination with DNA-PKi exerted a cooperative cytotoxic effect on the different types of cancer cells. These results suggest that combination treatment with DNA-PK and splicing inhibitors may represent a new therapeutic strategy. Moreover, our novel genetic map of vulnerabilities to DNA-PKi will serve as a valuable resource for those interested in DNA-PK function and therapy.

## 2. Results

### 2.1. Inhibition of DNA-PK Suppresses Cell Proliferation and Modulates mRNA Splicing

**Figure 1:**
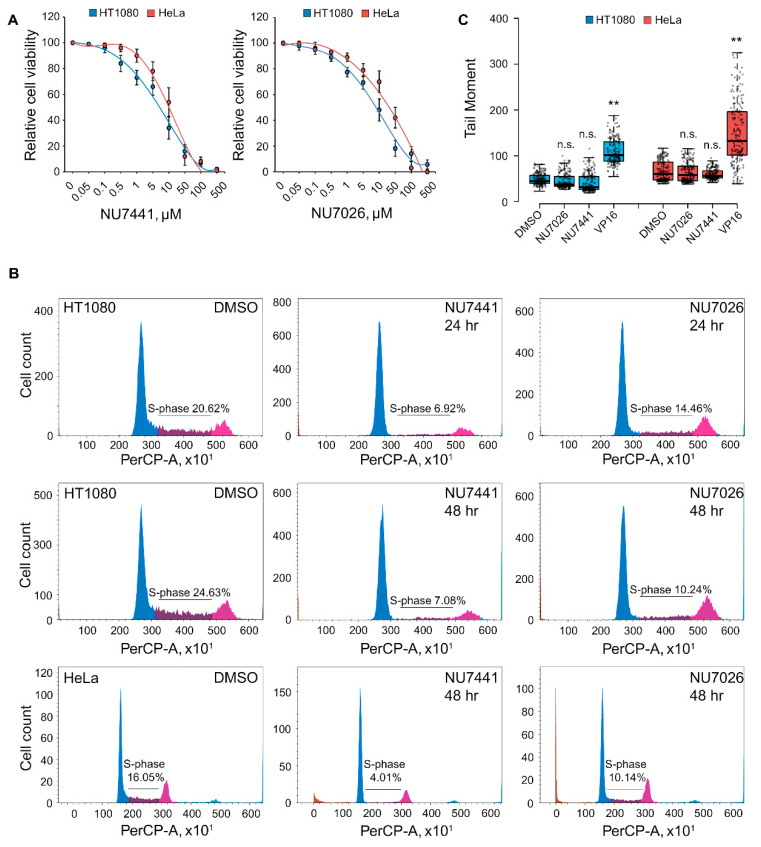
DNA-PK inhibition induces cell cycle arrest and cell death without DSBs. (**A**) Dose–response curves after treatment for HT1080 and HeLa cells with NU7441 or NU7026 at increasing concentrations for 4 days. Data are presented as mean ± SD (n = 3 biologically independent experiments); (**B**) analysis of cell cycle distribution of control HT1080 cells and cells treated with NU7441 (20 μM, 24–48 h) or NU7026 (50 μM, 24–48 h); (**C**) HT1080 and HeLa cells were treated with either DMSO, NU7441 (20 μM, 24 h), NU7026 (50 μM, 24 h), or DNA topoisomerase II inhibitor etoposide (VP16; 10 μg/mL, 1 h). A neutral comet assay was performed; the box plots show the tail moment. Horizontal lines represent the median. **, *p* < 0.01 by unpaired *t* test; n.s., not significant (n > 500).

We used human HT1080 and HeLa cells as a model to study the effects of pharmacological DNA-PK inhibition. The inhibition of DNA-PK activity using the highly specific pharmacological inhibitors NU7441 and NU7026 resulted in reduced cell viability (Figure 1A). Accordingly, cell cycle analysis showed that DNA-PK inhibition reduced the proportion of cells in S-phase. Thus, for HT1080 cells, the proportion of S-phase cells decreased by 3–3.5 and 1.5–2.5 times when treated with NU7441 or NU7026, respectively, after 24–48 h of exposure. A similar effect was observed for HeLa cells (Figure 1B). However, using a DNA comet assay, we did not observe a significant increase in the number of DSBs in cells after DNA-PKi treatment (Figure 1C). Therefore, the suppression of DNA-PK activity inhibits HT1080 and HeLa proliferation and cell cycle progression in the absence of DNA damage.

**Figure 2:**
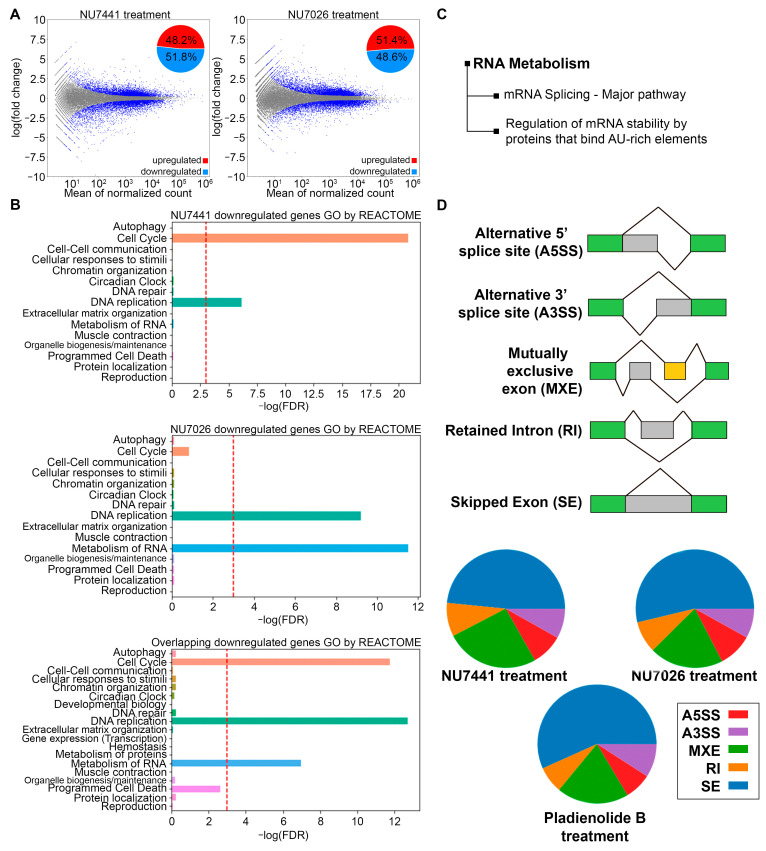
DNA-PK inhibition markedly affects the transcriptome and disrupts RNA splicing. (**A**) RNA-seq of HT1080 cell line treated with 20 μM NU7441 or 50 μM NU7026 in duplicate for 24 h before RNA was harvested. MA plots were generated for NU7441 or NU7026 treatment compared with control showing gene expression modulation with the number of transcripts upregulated (top) and downregulated (bottom). MA plot shows the logarithmic value of log fold change (fc; Y axis) and mean of normalized counts (X axis) for libraries. (**B**) GO term enrichment analyses were conducted using the RNA-seq data. The 15 most downregulated pathways were shown. Values were presented as the −log10 of FDR. (**C**) Visualization of the relationship in RNA metabolism GO terms using hierarchical clustering tree for the genes downregulated by both NU7441 and NU7026. (**D**) Diagrammatic representation of the statistically significant splicing alterations detected in response to DNA-PK inhibition. RNA-Seq data derived from HT1080 cells treated with either NU7441 (20 μM, 24 h), NU7026 (50 μM, 24 h), or pladienolide B (1.5 nM, 24 h) were analyzed for differential splicing activity. Events that passed an FDR ≤ 0.05, delta percent-spliced-in [dPsi] ≥ 0.05 were plotted in the pie chart.

Although these and previous findings link DNA-PK to the regulation of cell proliferation, and prior results demonstrate that tumor-associated DNA-PK promotes metastasis [20,21], the overall mechanisms by which DNA-PK affects disease progression are not completely understood. Therefore, to identify genes that may require DNA-PK kinase activity to promote cellular fitness, we sequenced the transcriptomes of HT1080 cells treated with DNA-PKi and found that both NU7441 and NU7026 significantly altered their transcriptional profiles. In both treatment groups, the number of downregulated genes slightly exceeded the number of upregulated genes (Figure 2A). The results of gene-level differential expression analysis can be found in the Supplementary Material (Table S1).

Using a false-discovery rate (FDR) threshold of ≤0.1, we analyzed Gene Ontology (GO) terms and found that the upregulated genes did not significantly cluster into GO terms following either NU7441 or NU7026 treatment. In contrast, GO term analysis of the downregulated genes revealed significant gene clustering in both the NU7441- and NU7026-treated groups (Figure 2B). Specifically, DNA-PK inhibition with NU7441 decreased the transcription of genes primarily related to the cell cycle and DNA replication, whereas treatment with NU7026 reduced the transcription of genes related to the cell cycle, DNA replication, and RNA metabolism. Of note, genes significantly downregulated by both NU7441 and NU7026 clustered into the same primary GO terms, including cell cycle, DNA replication, and RNA metabolism (Figure 2B). These findings demonstrate that DNA-PK affects transcriptional networks in HT1080 cells.

The involvement of DNA-PK in the regulation of the cell cycle and DNA replication has been well documented in previous studies. However, the role of DNA-PK in RNA metabolism is underexplored, prompting us to focus more closely on this group of genes. For the genes downregulated by both NU7441 and NU7026, we constructed a hierarchical clustering tree to find correlations between significant pathways (Figure 2C). We identified two main branches, including one with genes associated with mRNA splicing (e.g., *DDX46*, *HNRNPR, USP39, SART1*, *DHX15*, *SRSF3*, *KHSRP*, *SNRPA1*, *SNRPE*, *SF3B3*, *DHX8*, *DHX9*, *DDX23*, and *SNU13*, among others), and another with genes associated with the regulation of mRNA stability (e.g., *PARN*, *ELAVL1*, *EXOSC7*, *EXOSC5*, and *HNRNPD*, among others). The observation that gene sets associated with splicing regulation were affected by DNA-PK inhibition suggests that splicing activity is impaired in cells with reduced DNA-PK activity. To test this hypothesis, we treated cells with NU7441 or NU7026 and assessed global changes in splicing upon DNA-PK inhibition (Figure 2D). We identified 249,781 alternative splicing events induced by NU7026, 15,671 of which were statistically significant (FDR ≤ 0.05, delta percent-spliced-in [dPsi] ≥ 0.05), and 231,379 alternative splicing events caused by NU7441, 9987 of which were statistically significant (FDR ≤ 0.05, dPsi ≥ 0.05). This result equates to splicing alterations in 4542 and 5765 genes upon treatment with NU7441 and NU7026, respectively. Exon skipping was the most commonly observed alteration (Figure 2D). Interestingly, the splicing changes induced by NU7441 and NU7026 were similar to those caused by pladienolide B, an inhibitor of the SF3B1 subunit of the spliceosome, which induced 25,239 statistically significant alternative splicing events (FDR ≤ 0.05, dPsi ≥ 0.05) out of 337,202 identified (Figure 2D).

### 2.2. Co-Targeting of DNA-PK and Splicing Induces Synergistic Anti-Proliferative Effects in Human Cancer Cells

**Figure 3:**
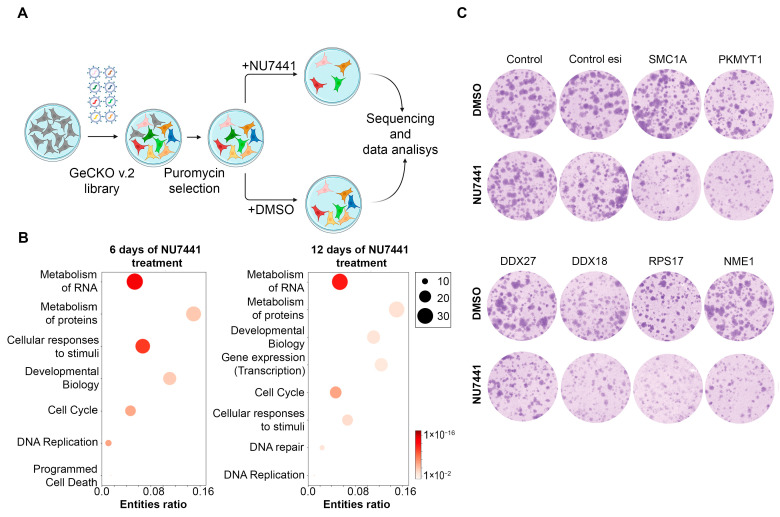
Identification of mutations that sensitize cells to DNA-PK inhibition. (**A**) Schematic of genome-wide CRISPR/Cas9 screening workflow. (**B**) Gene Ontology (GO) term enrichment analysis using Reactome database of genes that were hits in screens at times points T6 and T12 (FDR ≤ 0.2). Circle size indicates the number of genes from the core set included in each GO term, color indicates a negative log *p*-value, and *x*-axis position indicates the fold enrichment compared to the whole genome reference set. (**C**) Cell growth assays were performed using HT1080 cells. The cells were transfected with the indicated esiRNAs, and after 24 h, 4 μM NU7441 was added for 14 days. Images of colonies in colony formation assay are presented.

As previously described, NU7441 and NU7026 suppressed cell proliferation and cell cycle progression in HT1080 cells. However, this effect was relatively modest and only occurred following treatment with relatively high concentrations, indicating some level of cell resistance to these inhibitors. Therefore, to identify genes that promote DNA-PKi resistance, we conducted CRISPR/Cas9 somatic genetic screens. The screens, schematically depicted in Figure 3A, were performed essentially as previously described [22].

Specifically, Cas9-expressing HT1080 cells were transduced with a lentiviral library of single-guide (sg) RNAs; after selection and editing, the resulting pool of gene-edited cells were split into two populations. One population (the control) was incubated with DMSO for the duration of the screen, while the second population was incubated with a sublethal dose of NU7441 that killed approximately 20% of the cells. The abundance of sgRNAs was determined in each population by sequencing after 6 (T6) or 12 (T12) days of treatment, and gene depletion was assessed using the latest version of the drugZ tool [23]. The results of gene-level differential expression analysis can be found in the supplementary material (Tables S2 and S3). Using a hit-selection threshold with FDR ≤ 0.2, we identified 96 and 124 genes at T6 and T12, respectively, that promoted DNA-PKi resistance in HT1080 cells treated with NU7441. GO term enrichment analysis using the Reactome pathway database showed that gene sets related to the cell cycle, metabolism of protein and RNA, DNA replication, and cellular response to stimuli were significantly enriched at both T6 and T12 (Figure 3B). The cell cycle GO term included regulators of cyclin-dependent kinases such as *CCNA2* and *PKMYT1*, as well as regulators of the G2/M transition, microtubule-dependent processes, and chromosome separation (*CDC20*, *CDC23*, *TUBGCP3*, *TUBGCP6*, *HAUS7*, and *HAUS8*). This group also included numerous genes encoding proteasome subunits (*PSMA2*, *PSMB2*, *PSMB5*, *PSMD7*, and *PSMD14*). The DNA replication GO term included functional components of the replisome, such as *PCNA*, *GINS3*, *PRIM1*, and *RFC5*. The groups of genes associated with protein metabolism and the cellular response to stimuli predominantly encoded ribosomal (e.g., *RPL18A*, *RPS3*, *RPL35*, *RPL38*, *RPL18*, *RPS15A*, *RPS19*, *RPL18A*, and *RPS17*, among others) or proteasomal (e.g., *PSMB5*, *PSMD7*, *PSMB2*, *PSMA2*, and *PSMD14*, among others) proteins.

Next, we aimed to directly confirm that the genes identified in the CRISPR/Cas9 screen indeed modulate the response to DNA-PKi. To this end, we randomly selected several targets with significant FDR values (*SMC1A*, *PKMYT1*, *DDX27*, *DDX18*, *RPS17*, and *NME1*); reduced their expression levels in HT1080 cells, which were used in the initial screen, using RNA interference; and analyzed cell viability in the presence of NU7441. Knockdown efficiency was monitored by RT-PCR (Figure S1); expression was generally reduced 7–10-fold. Using a clonogenic assay, we observed a marked increase in cell sensitivity to NU7441 following the knockdown of each selected gene compared to a scrambled control (Figure 3C). These results indicate that all of the examined genes contribute to NU7441 resistance, thereby validating the results of our screen.

**Figure 4:**
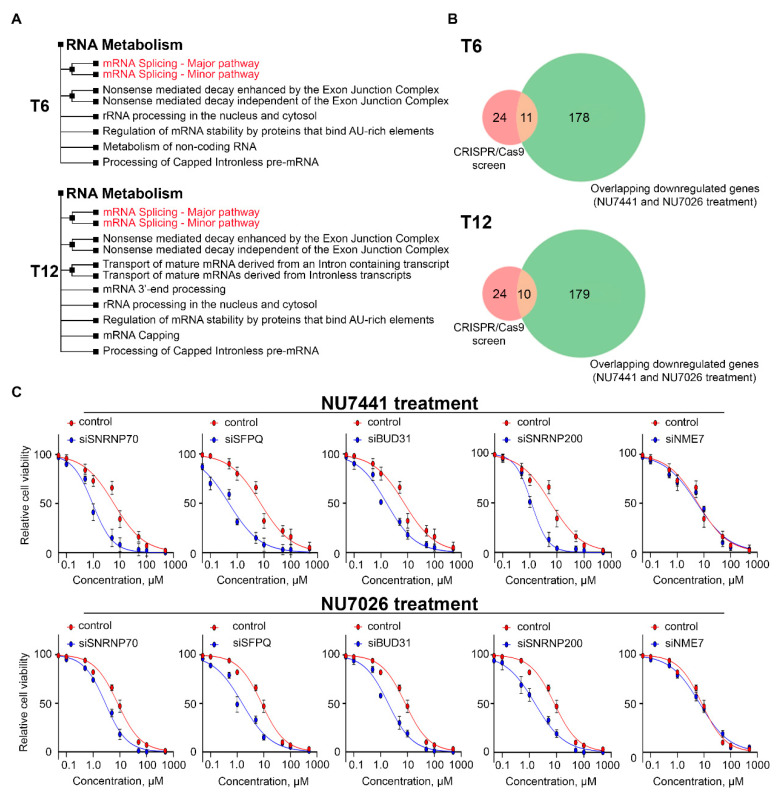
Disruption of RNA splicing increases cell sensitivity to DNA-PKi. (**A**) Visualization of the relationship in RNA metabolism GO terms using hierarchical clustering tree for genes that were hits in CRISPR/Cas9 screening at times points T6 and T12. (**B**) The genes associated with RNA metabolism were overlapped between the data from genome-wide CRISPR/Cas9 screening and RNA-seq. (**C**) Dose–response curves after treatment with NU7441 or NU7026 at increasing concentrations for HT1080 cells with siRNA-mediated gene knockdown as indicated. Data are presented as mean ± SD (n = 3 biologically independent experiments).

Next, we sought to find genes associated with RNA splicing from among those identified in the CRISPR/Cas9 screen. To this end, we focused on the RNA metabolism-associated GO term, which was also the most significantly enriched term at both T6 and T12. A hierarchical clustering tree for this term revealed the enrichment of genes involved in mRNA processing and splicing (*SFPQ*, *DHX15*, *SNRPE*, *SF3A3*, *PPIL2*, *SNRNP200*, *SNRPC*, *RAE1*, *SNRPA1*, *SNRNP70*, *PCF11*, *BUD31*, *CPSF3*, *CPSF2*, and *LENG1*), as well as rRNA processing and ribosome biogenesis (*NOP2*, *NIP7*, *NOL6*, *RPL17*, *RPL18*, *RPL30*, *RPS15A*, and *RPS26*) (Figure 4A). It is also important to note that some of these genes overlapped with the downregulated genes from the RNA-seq analysis (Figure 4B). Therefore, we hypothesized that defects in genes associated with RNA metabolism may synergistically enhance the effect of DNA-PKi, which reduces the transcription of genes within the same metabolic pathways.

We next aimed to validate the significance of the splicing-related genes identified in the CRISPR/Cas9 screen. To this end, we selected several targets from this group with the lowest FDR values and for which DNA-PKi did not affect transcription. The genes chosen were *SFPQ* (FDR = 0.0005), *SNRNP70* (FDR = 0.074), *SNRNP200* (FDR = 0.01), and *BUD31* (FDR = 0.01). Gene *NME7* (FDR = 0.996) was selected as a negative control, as it had one of the highest FDR values among all hits in the CRISPR/Cas9 screen. Using RNA interference, we individually downregulated the expression of the selected genes in HT1080 cells and analyzed cell viability in the presence of DNA-PKi. Knockdown efficiency was monitored by RT-PCR (Figure S1). We observed a significant increase in cell sensitivity to NU7441 or NU7026 following the knockdown of each of these genes (Figure 4C). In contrast, *NME7* knockdown did not elicit a significant effect.

**Figure 5:**
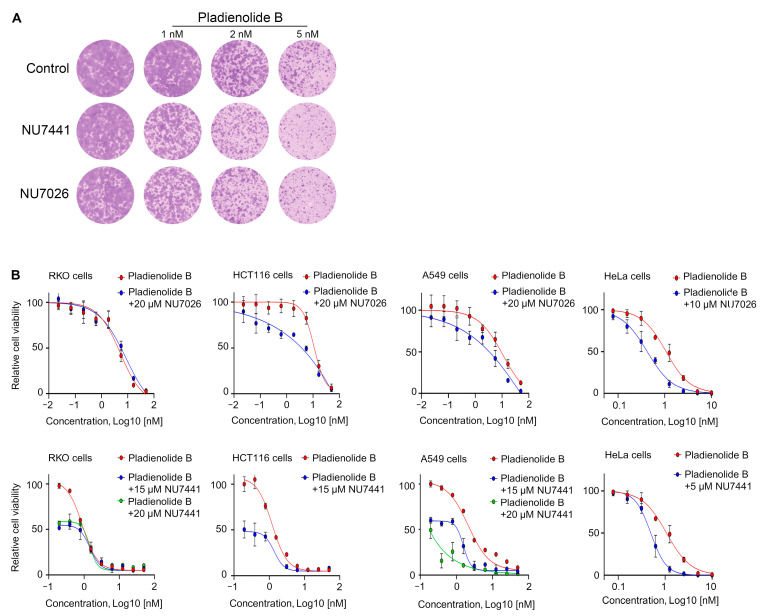
Pladienolide B-mediated splicing inhibition enhances the cytotoxicity of DNA-PKi across various cancer cell types. (**A**) Cell growth assays were performed using HT1080 cells. The cells were seeded and treated after 24 h with 1–5 nM pladienolide B. After 24 h, cells were washed, and 4 μM NU7441 or 5 μM NU7026 was added. Cells were then incubated for 14 days. Images of colonies in colony formation assay are presented. (**B**) RKO, HCT116, HeLa, and A549 cells were treated with pladienolid B at increasing concentrations for 24 h. Subsequently, the cells were washed, and DNA-PKi was administered at the indicated concentrations for an additional 3 days. Dose–response curves are presented. Data are presented as mean ± SD (n = 3 biologically independent experiments).

Finally, we decided to check whether the cytotoxicity of DNA-PKi could be enhanced by combining them with splicing inhibitors. To do this, we pretreated HT1080 cells with pladienolide B, an inhibitor of the splicing factor SF3b, and then additionally incubated the cells with NU7441 or NU7026. Using a clonogenic assay, we confirmed that compromising mRNA splicing via pladienolide B (SF3b inhibitor) treatment also significantly increased HT1080 cell sensitivity to NU7441 or NU7026 (Figure 5A). Lastly, we sought to evaluate the effects of combining splicing inhibitors with DNA-PKi in additional tumor-specific cell lines. For these experiments, we employed the A549 human alveolar carcinoma cell line, HeLa human cervical carcinoma cell line, and HCT116 and RKO human colorectal carcinoma cell lines. Our results demonstrate that treatment with NU7441 significantly potentiated the cytotoxic effects of pladienolide B in A549 and HeLa cells, with a comparatively moderate effect observed in HCT116 cells (Figure 5B). Likewise, NU7026 enhanced the cytotoxicity of pladienolide B in A549, HeLa, and HCT116 cell lines; however, neither NU7441 nor NU7026 exhibited any enhancement of cytotoxic effects of pladienolide B in RKO cells (Figure 5B).

Collectively, our results suggest that DNA-PK may play a crucial role in the regulation of mRNA splicing and that its inhibition may induce synthetic lethality in the context of splicing machinery-encoding gene deficiencies or impaired splicing regulation. Furthermore, our findings indicate that combination treatment with DNA-PKi and mRNA splicing inhibitors may exert a cooperative cytostatic effect on some cancer cell types.

## 3. Discussion

DNA-PK is overexpressed, hyperactivated, and drives aggressive phenotypes in certain cancer types [24]; however, the underlying mechanisms are not fully understood. Our study identified DNA-PK as a transcriptional regulator of numerous established and novel cancer-associated pathways in the absence of exogenous DNA damage. Specifically, GO-term analysis of the HT1080 cell transcriptome following DNA-PKi treatment revealed well-established pathways modulated by DNA-PK, including DNA replication, cell cycle, and proliferation pathways [6]. Additionally, our study identified mRNA splicing as a novel DNA-PK-regulated process. In particular, we found that DNA-PK inhibition reduced the expression levels of genes within the primary splicing pathway and elicited alternative splicing events for more than 4000 genes, an effect comparable to that of direct spliceosome inhibitors.

An intriguing question raised by our observations is how DNA-PK might mechanistically influence pre-mRNA splicing. Several potential scenarios can be envisioned. The most straightforward scenario is that DNA-PK positively regulates the transcription of splicing-associated genes, as demonstrated in our study. In an alternative model, DNA-PK may control the phosphorylation status of splicing factors. This idea is consistent with earlier observations that DNA-PK can translocate to splice speckles upon genotoxic DNA damage [25] and phosphorylate several proteins of heterogenous nuclear RNP [26] and ribosomal proteins [17], which participate in various aspects of RNA metabolism. In this context, it is noteworthy that both the activity and subcellular distribution of many spliceosome-associated splicing factors are regulated by phosphorylation [27]. In a third scenario, DNA-PK may physically bind RNA, potentially affecting the activity of splicing machinery independently of its kinase function. This scenario could be analogous to how DNA-PK operates at sites of DNA damage in the NHEJ pathway. Specifically, the kinase activity of DNA-PK is only crucial for autophosphorylation and its subsequent dissociation from DNA damage sites [28], not for the recruitment and activation of downstream repair factors [29]. Thus, it has been suggested that DNA-PK may serve as a physical scaffold that facilitates the proper alignment of broken DNA ends during NHEJ. In this regard, it is interesting to note that DNA-PK can indeed bind a wide range of cellular RNAs—including the U3 small nucleolar RNA, which is critical for efficient ribosomal RNA processing [18]—via complex formation with the KU heterodimer.

Using CRISPR/Cas9 screening, we identified splicing genes that contribute to cellular resistance to DNA-PK inhibition. We demonstrated that cells deficient in splicing factors such as *SFPQ*, *SNRNP70*, *SNRNP200*, and *BUD31* become significantly more sensitive to DNA-PK inhibition. Furthermore, we showed that combination treatment with a splicing inhibitor (pladienolide B, an SF3b inhibitor) and a DNA-PKi (NU7441 or NU7026) exerted a synergistic cytotoxic effect across multiple cancer cell lines, including HT1080, HeLa, A549, and HCT116. Notably, RKO cells exhibited resistance to the combined treatment of pladienolide B and DNA-PKi, highlighting the differential sensitivities of various cancer cell types to this therapeutic approach. This resistance is likely attributable to variations in DNA-PK expression levels, activity, and its interactions with other genes and signaling pathways across different tumor types.

DNA-PKi and splicing inhibitors represent promising anticancer agents [30,31,32,33]; however, clinical trials have demonstrated limitations in their clinical applicability. For example, the administration of splicing inhibitors at therapeutic doses is hindered by significant side effects [34,35,36], whereas DNA-PKi generally show the greatest efficacy as radio- and chemosensitizers [37,38,39,40,41]. Our findings demonstrate that the combination of pladienolide B, a splicing inhibitor, with DNA-PKi synergistically enhances their cytotoxic effects on cancer cells, indicating greater therapeutic potential compared to monotherapy. Although this study is limited to in vitro models, it underscores the importance of further investigating the effects of inhibitors of splicing and DNA-PKi in tumor models. Employing patient-derived tumor organoid models or patient-derived xenograft (PDX) models could provide a more accurate representation of tumor biology and help elucidate the therapeutic potential of this drug combination, thereby increasing the likelihood of clinical translation.

## 4. Materials and Methods

### 4.1. Cell Culture

Human HT1080 (ATCC^®^CCL-121™), HEK293T (ATCC^®^CRL-3216™), HeLa (ATCC^®^CCL-2™), HCT116 (ATCC^®^CCL-247™), RKO (ATCC^®^CRL-2577™), and A549 (ATCC^®^CCL-185™) cells were cultured in Dulbecco’s Modified Eagle Medium (DMEM), high glucose media (PanEco, Moscow, Russia; C415) supplemented with 10% fetal bovine serum (Hyclone, Logan, UT, USA; SV30160.03) and 1% penicillin–streptomycin. The cells were cultured at 37 °C in a conventional humidified CO_2_ incubator.

In the DNA-PK inhibition experiments, cells were exposed to NU7441 (Sigma-Aldrich, St. Louis, MO, USA; SML3923) or NU7026 (Adooq Bioscience, Irvine, CA, USA; A12752) at defined concentrations and time points. For splicing inhibition assays, cells were treated with pladienolide B (Santa Cruz, Dallas, TX, USA; #445493-23-2) at the specified concentrations and incubation periods.

### 4.2. CRISPR Screen

For the CRISPR screen, the human knockout pooled library GeCKO V2 (Addgene, #1000000048) was used. This library comprises 123,411 single-guide RNAs (sgRNAs) that target 19,050 genes. GeCKO V2 gRNAs were amplified and packaged into lentiviruses using the HEK293T cell line and concentrated to increase viral titer. In total, 170 millions of HT1080 cells were infected at a multiplicity of infection (MOI) of 0.3 to obtain a 400x coverage and were selected during 3 days at 1 μg/mL of Puromycin (Sigma, P7255). Next 20 × 10^6^ cells were treated with dimethyl sulfoxide (DMSO, Sigma, #472301), and 20 × 10^6^ cells were treated with 2.5 μM of NU7441. Cells were collected for DNA extraction at day 0 (T0), day 6 (T6), and day 12 (T12). Frozen cell pellets were thawed, and genomic DNA was extracted with a Quick-DNA Midiprep Plus Kit (Zymo Research, Irvine, CA, USA; D4075) according to the manufacturer’s protocol. Polymerase chain reaction (PCR) was performed according to the instructions in the original paper [22].

Next-generation sequencing (NGS) was performed on the Illumina NextSeq. Raw reads were analyzed using MAGeCK ver.0.5.9.5 (https://github.com/liulab-dfci/MAGeCK) and drugZ ver.1.1.0.2 build 116 (https://github.com/hart-lab/drugz). Briefly, count files were generated using the mageck count function and Human GeCKOv2 Library list. The corresponding result files for each selected time point were then subjected to drugZ with an unpaired option. Synergistic/synthetic lethal genes were selected using a false discovery rate (FDR) ≤ 0.2 and a normZ score ≤ −3. We annotated the synthetic lethal genes for time points T6 and T12 for both DMSO-treated and NU7441-treated cells relative to T0. To exclude inherently lethal genes from the final list, we removed overlapping genes between DMSO-treated and NU7441-treated samples at the corresponding time points.

### 4.3. RNA Interference

The transfection of cells with specific small interfering RNAs (siRNAs) or endoribonuclease-prepared siRNAs (esiRNAs) was carried out using Lipofectamine 3000 (Thermo Fisher, Waltham, MA USA; L3000075) transfection reagent according to the manufacturer’s instructions. The cells were transfected with 30 nM SNRNP70 siRNA (5′- CACGCAGAUGGCAAGAAGA; 5′- UGGUCUACAGUAAGCGGUCAGGAAA; 5′- CAAUCAACCUUAUUGUGGCAUUGCG), 30 nM BUD31 siRNA (5′-GCAGAGAACUCUAUGAAUA; 5′-CAAGAAUCCAAGCCUGCAA; 5′-CCUCUGUUCUCGAUUACCUUGGCAA), 30 nM SNRNP200 siRNA (5′-GGACGAGCACCUCAUUACA; 5′-AGAAGAGCCAAGCGAAGAA; 5′-CAGGCCCUGUCAUUGCGCCUCUCUU), 30 nM NME7 siRNA (5′-GAGAUGAUGCUAUAUGUGA; 5′-GAUCAACAAGAGCACUGUA; 5′-CCCUGGAACUCUCAGAGCAAUCUUU), 100 nM SFPQ/PSF siRNA (Santa-Cruz, #sc-38304), 200 ng/mL nM SMC1A esiRNA (Sigma, #EHU074001), 200 ng/mL PKMYT esiRNA (Sigma, #EHU149711), 200 ng/mL DDX27 esiRNA (Sigma, #EHU049171), 200 ng/mL DDX18 esiRNA (Sigma, #EHU105171), and 200 ng/mL RPS17 esiRNA (Sigma, #EHU112101). Experiments were usually performed 48–72 h after transfection.

### 4.4. Cell Viability Assay

Control HT1080 cells or cells with gene knockdown were seeded in 96-well plates at a density of 1500 cells per well in 100 μL cultural media overnight. The adhered cells were treated with DNA-PKi as indicated for 5 days. HCT116, A549, and RKO cell lines were seeded in 96-well plates at a density of 5000 cells per well in 200 μL of medium and incubated overnight to allow for adherence. Following incubation, adherent cells were treated with pladienolide B at the specified concentrations for 24 h. Subsequently, the cells were washed, and DNA-PKi were administered at the indicated concentrations for an additional 3 days. Cell viability assays were performed using alamarBlue (ThermoFisher Scientific, DAL1025) according to the manufacturer’s instruction. A dose–response curve was used to assess drug response.

### 4.5. Clonogenic Survival Assay

For the assessment of clonogenic survival, HT1080 cells were seeded in 6-well plates and allowed to adhere for 24 h prior to treatment. Subsequently, the cells were exposed to the compounds NU7441 or NU7026. Following drug administration, the cells were incubated for a period of 14 days to allow colony formation. In the case of combination treatment, pladienolide B was added 24 h post seeding. After an additional 24 h, the cells were washed, and either NU7441 or NU7026 was introduced to the culture. The incubation continued for a further 14 days. Colonies were then fixed using methanol and stained with Giemsa dye (PanEco, O080) to facilitate enumeration.

### 4.6. Cell Cycle Assay (FACS)

HT1080 or HeLa cells were seeded in 6-well plates and treated with the compounds for 24–48 h. Next the cells were trypsinized with 0.25% trypsin for several minutes at 37 °C. The trypsin was inactivated with a 4-fold volume of culture medium. Cell sediments were treated with a PI buffer (0.1% sodium citrate, 0.3% NP-40, 50 mg/mL propidium iodide, 50 mg/mL RNaseA) for 30 min in the dark. After washing, the samples were analyzed using a CytoFLEX S Flow Cytometer (Beckman Coulter Brea, CA, USA).

### 4.7. RNA Sequencing (RNA-Seq)

HT1080 cells were seeded in 12-well plates at appropriate numbers to allow cells to grow to ∼90% confluence at the endpoint. For inhibitor treatment, cells were seeded 24 h before being treated with vehicle control (0.1% DMSO) or indicated chemicals at the stated concentrations and time periods (up to 2 days). Total RNA was isolated and purified using TRIzol reagent (Thermo Fisher Scientific, 15596026) according to the manufacturer’s instructions. cDNA libraries for sequencing were prepared using the NEBNext^®^ Poly(A) mRNA Magnetic Isolation Module (New England Biolabs, Ipswich, MA, USA; E7490S) according to the manufacturer’s protocol. Next-generation sequencing (NGS) was performed on the Illumina NextSeq targeting 50 millions of reads per sample.

RNA-seq reads were mapped to the reference human genome hg38 assembly using STAR 2.7.10a_alpha_220314 with the following parameters: --outFilterType BySJout --outFilterMmkltimapNmax 20 --alignSJoverhangMin 8 --alignSJDBoverhangMin 1 --outFilterMismatchNmax 999 --outFilterMismatchNoverReadLmax 0.04 --alignIntronMin 20 -- alignIntronMax 1000000 --alignMatesGapMax 1000000. The resulting SAM files were then sorted and converted to BAM format using samtools v 1.3.1.:

1. For differential expression analysis, read counts for transcripts were obtaining using featureCounts and the NCBI RefSeq gtf file (downloaded from UCSC) with the following parameters: -F GTF -M -s 0 -p -B -C -T 25 –countReadPair. The resulting raw count files were subjected to R package DESeq2.
2. Alternative splicing analysis was performed using rMATS-turbo v 4.3.0 (https://github.com/Xinglab/rmats-turbo) with the following parameters: -t paired --libType fr-unstranded --readLength 150 --nthread 10 --novelSS --individual-counts and GENECODE v45 annotation. The resulting rMATS-turbo files were processed using a custom python script. An alternative splicing event was considered significant if its FDR ≤ 0.05 and dPSI ≥ 0.05. dPSI represents the difference between PSI values for two samples. PSI (Percent Spliced In) represents the percentage of transcripts that include a specific exon or splice site, calculated from RNA-seq read counts supporting specific exons or splice junctions, normalized by the effective lengths of distinct transcript isoforms (e.g., exon inclusion versus skipping).

### 4.8. Neutral Comet Assay

After treatment, cells were trypsinized with 0.25% trypsin for several minutes at 37 °C. The trypsin was inactivated with a 4-fold volume of DMEM. Cell suspension at a concentration of 10^5^ cells/mL was mixed in a 1:1 ratio with LMAgarose (Trevigen, #4250-050-02) at 37 °C. The mixture was pipetted onto comet slides (Trevigen, Gaithersburg, MD, USA; #3950-300-02) that had been pre-coated with a 1% normal melting point agarose (BioinnLabs, Rostov-on-Don; Russia; #9012-36-6) base layer. The drop containing the cells was covered with a glass cover slip and incubated at 4 °C for 5 min. After incubation, the cover slips were removed, and the slides were immersed in lysis solution (30 mM ethylenediaminetetraacetic acid (EDTA), 0.5% sodium dodecyl sulfate (SDS), and 10 mM Tris–HCl, pH 8.0, supplemented with 500 µg/mL proteinase K) and incubated at 37 °C for 1 h. After lysis, the slides were washed three times for 5 min in PBS and incubated in 1× TBE (Tris-Borate-EDTA buffer) for 20 min at 4 °C. Electrophoresis was performed in a Trevigen electrophoresis system (Trevigen, #4250-050-ES) for 10 min at 4 °C and 1 V/cm in 1× TBE. The comets were counterstained with SYBR Green for 1 h (1:3000; Thermo Scientific, #S7563). The comets were visualized at four-times magnification using an inverted Nikon Eclipse Ti-E fluorescence microscope equipped with a Nikon Intensilight C-HGFI light source (objective: Nikon Plan Fluor 4/0.13; camera: DS-Qi2). The images of the comets were analyzed using CellProfiler software (version 2.1.1 rev 6c2d896).

### 4.9. Gene Expression Analysis

RNA was extracted from cells using TRIzol reagent (Life Technologies, Carlsbad, CA, USA; #15596018). All RNA samples were further treated with DNase I (Thermo Fisher Scientific, #EN0521) to remove the residual DNA. RNA (1 μg) was reverse-transcribed in a total volume of 20 μL for 1 h at 42 °C using 0.4 μg of random hexamer primers (Fermentas, Waltham, MA, USA; #SO142) and 200 U of reverse transcriptase (Thermo Fisher Scientific, EP0441) in the presence of 20 U ribonuclease inhibitor (Thermo Fisher Scientific, EO0381). The cDNA obtained was analyzed by qPCR using a CFX96 Touch Real-Time PCR detection system (Bio-Rad Laboratories, Hercules, CA, USA). PCRs were performed in 20 μL reaction volumes that included 50 mM Tris-HCl (pH 8.6), 50 mM KCl, 1.5 mM MgCl_2_, 0.1% Tween 20, 0.5 μM of each primer, 0.2 mM of each deoxynucleoside triphosphate, 0.6 μM EvaGreen (Biotium, Fremont, CA, USA; #31000), 0.2 U Hot Start Taq Polymerase (SibEnzyme, Novosibirsk, Russia; B309), and 50 ng cDNA. Primers used in this study are listed in Table S4. Levels of mRNA normalized to GAPDH mRNA are shown in the corresponding figures.

## Data Availability

All data are available in the Supporting Information. Sequencing raw read files are available upon request.

## Supplementary Materials

The following supporting information can be downloaded at https://www.mdpi.com/article/10.3390/ijms252111810/s1.
