# Supplementary material for: Disruption of RNA Splicing Increases Vulnerability of Cells to DNA-PK Inhibitors
Source: Int J Mol Sci. 2024 Nov 3;25(21):11810. doi: 10.3390/ijms252111810 (PMC11546466; doi:10.3390/ijms252111810)
Supplement: Supplementary file 1 [file ijms-25-11810-s001.zip › Figure S1_Evaluation of knockdown efficiency for the target genes.pdf]

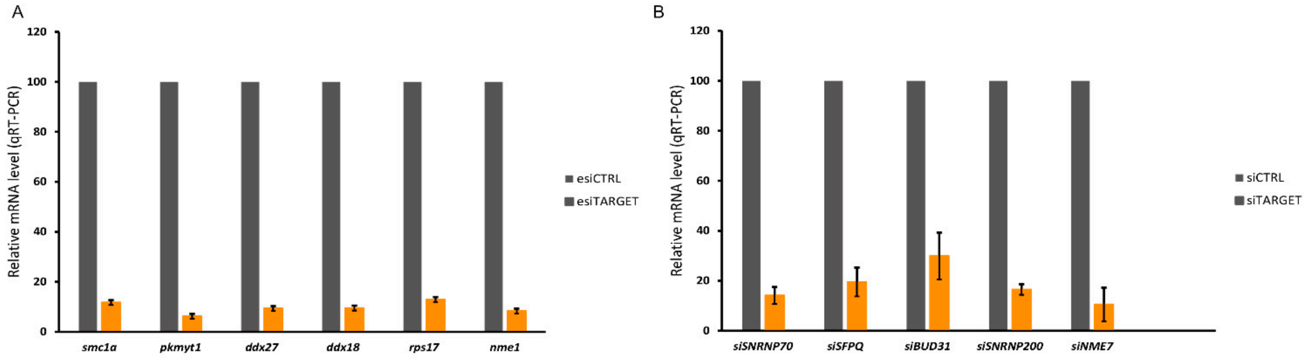

Figure S1. Evaluation of knockdown efficiency for the target genes. **(A)** Quantitative reverse-transcription PCR (qRT-PCR) analysis of SMC1, PKMYT1, DDX27, DDX18, RPS17 and NME1 mRNA levels in human HT1080 cells transfected with the corresponding esiRNA sets.The amplification levels of each kinase cDNA were normalized to the amplification level of GAPDH cDNA. Data are presented as mean ± SD (n = 3 biologically independent experiments). **(B)** Quantitative reverse-transcription PCR (qRT-PCR) analysis of SNRNP70, SFPQ, BUD31, SNRNP200 and NME7 mRNA levels in human HT1080 cells transfected with the corresponding siRNA sets. The amplification levels of each kinase cDNA were normalized to the amplification level of GAPDH cDNA. Data are presented as mean ± SD (n = 3 biologically independent experiments).
